# Techniques of repair, the circulation of knowledge, and environmental transformation: Towards a new history of transportation

**DOI:** 10.1177/00732753211046450

**Published:** 2021-09-28

**Authors:** Dániel Margócsy, Mary Augusta Brazelton

**Affiliations:** University of Cambridge, UK; University of Cambridge, UK

**Keywords:** Circulation of knowledge, history of repair, history of technology, transportation technology, maintenance

## Abstract

It is the aim of this article to put questions of maintenance and repair in the history of science and technology under scrutiny, with a special focus on technologies and methods of transportation. The history of transportation is a history of trying to avoid shipwrecks and plane crashes. It is also a history of broken masts, worm-eaten hulls, the flat tires of cars, and endless delays at airports. This introductory article assesses the technological, scientific, and cultural implications of repairing and maintaining transportation networks. We argue that infrastructures for maintenance and repair played just as important a role in the history of transportation as the wharves and factories where ships, cars, trains, and airplanes were originally built. We also suggest that maintenance and repair are important sites of knowledge production, and a historical account of these practices provides a new, decentered narrative for the development of modern science and technology.

## Introduction

The philosophical paradox of the ship of Theseus asks what happens if a ship is exhibited in a museum, and its rotting pieces are replaced by replicas one by one, until no plank remains from the original construction. Is the exhibited object still the same as the original, or is it something entirely new?

It is not an accident that this abstract philosophical question was first posed about ships. Ancient and early modern ships, like twenty-first-century cars or airplanes, were constantly rebuilt, and it is anyone’s guess what remained constant beyond the name. It is one of the paradoxes of transportation that although vehicles facilitate the movements of goods, people, and ideas across the globe, they themselves do not stay the same across many voyages. Consider the *Avondster*, for instance – an East Indiaman sunk in the Bay of Galle near Sri Lanka in the mid-seventeenth century. It was originally built in England, using local elm wood. After it was captured by the Dutch, its hull received double planking in Asian waters and its anchor was remade from non-European timber.^1^ By the time it had gone down, many of the *Avondster*’s parts had been replaced and repaired by skilled craftsmen across the world. Similar stories could be told of the twenty-first-century automobile and flight industry, which anticipate the need for constant repairs and part replacement. The size of the aircraft maintenance and repair business, for instance, is comparable to that of airplane production itself.^2^

It is the aim of this special issue to put questions of maintenance and repair in the history of science and technology under scrutiny, with a special focus on technologies and methods of transportation. The history of transportation is a history of trying to avoid shipwrecks and plane crashes. It is also a history of broken masts, worm-eaten hulls, the flat tires of cars, and endless delays at airports. This special issue assesses the technological, scientific, and cultural implications of repairing and maintaining transportation networks. We argue that infrastructures for maintenance and repair played just as important a role in the history of transportation as the wharves and factories where ships, cars, trains, and airplanes were originally built. We also suggest that maintenance and repair are important sites of knowledge production, and a historical account of these practices provides a new, decentered narrative for the development of modern science and technology. As Cátia Antunes has recently emphasized, a focus on repair techniques in transportation history offers the opportunity to examine how non-European peoples and materials played an essential role in the development of large-scale technological enterprises.^3^

Historians of science and technology have long preferred to study the emergence of new technologies, instead of examining the survival and maintenance of older ones. Yet, as David Edgerton has reminded us, old technologies have prevailed for much longer than what one could predict from reading such triumphalist accounts.^4^ Historians such as Jessica Meyerson, Andy Russell, and Lee Vinsel have subsequently begun to explore how maintenance and repair are crucial elements of the history of technology.^5^ In the field of human and especially urban geography, the work of Stephen Graham and Nigel Thrift has engineered a similar growth of interest, examining how issues of repair and infrastructure shed light on hidden inequalities in the neoliberal global order.^6^ As these authors emphasize, technological repair is unpredictable and requires complex expertise: yet it often also relies on makeshift, bricolaged solutions to cope with breakdowns.

The historiography of transportation has generally neglected to give attention to such breakdowns, instead traditionally focusing on inventions and constructions like the establishment of canal systems, the laying of Macadam roads, the development of steam ships, and the formation of railways.^7^ The same approach pervades more recent histories of mobility and globalization that emphasize how inventions in mobility quickly spread across the world, presuming that the modern technologies that transform society run without needing much investment in their repair.^8^ Of course, this is rarely, if ever, the case. Throughout history, a variety of different strategies have been developed for travelers to deal with issues of repair arising at distant locations, with important ecological and political consequences. We suggest that the history of science and technology would benefit greatly from attention to the ways in which different modes of transportation require different types of maintenance.

This special issue argues that repair in transportation matters for three different reasons, to be elaborated below. First, ships, cars, pack animals, and airplanes travel *by definition*. Their sites of repair are not at the sites of their construction. The materials and know-how involved in fixing and repairing these technologies, therefore, need to travel together with the thing itself or have to be created anew at a new site. Yet as sociologists of science have long argued, the transplantation of knowledge requires complex political and social coordination, and it raises concerns about the geographies of expertise and natural resources, as well.^9^ The movement of knowledge about repair is not, then, simply a subsidiary function of the movement of information about ships and other transportation technologies. It travels in a way that is distinct from other forms of knowledge. Second, although recent scholarship on the circulation of scientific knowledge has emphasized the importance of travel and cross-cultural contact for the development of natural knowledge, it has not systematically considered how the material conditions of transportation affect the way knowledge develops on the road – or even how knowledge might fail to circulate at all, because of problems of transportation.^10^ More work needs to be done to demonstrate how transportation conditions what kinds of knowledge are produced in cross-cultural exchanges. Third, a focus on repair helps us reorient and connect the literature on theories of modernity and environmental history. It reveals how increases in global connectivity may often result in the exploitation of local expertise and the depletion of natural resources across the globe. Travel significantly shapes what humans know about their natural environment, even as it destroys that environment. The detailed studies of transport technologies provided by the contributions of this issue make a powerful argument for the significance of movement and displacement, and attendant issues of maintenance and repair, to current narratives of the history of science and technology.

### Repair strategies in the longue durée

Crashes and wrecks have always featured in the history of long-distance travel across the ages. Throughout the centuries, travelers developed a variety of strategies of maintenance and repair to ensure they reached their destination and then came back. Here, we survey the strategies of repair that have characterized technologies of transportation from the early modern period to the present day. Some travelers relied on multiple vessels or vehicles, hoping that at least one would make it through. A second, less costly alternative was to bring extra parts and skilled craftsmen on one vessel in order to rely on tried and trustworthy procedures of repair. Last, but not least, travelers could also decide to wing it, so to speak: to deal with repairs along the way, hoping to find materials and skilled workers at distant locations. These three strategies exploited natural and human resources in different ways, and had divergent impacts on the environment, local populations, and the development of natural knowledge.

As the accounts of Christopher Columbus’ first voyage emphasize, exploratory travel in the age of sail often depended on taking several vessels on a voyage together at the same time.^11^ If one ship broke down or sank, the others could carry the crew and the contents of the hold onwards. The strategy of sending out multiple vessels on such voyages was based upon the economics of waste, accepting that ships, and especially sailors, were disposable goods that could be replaced. By the nineteenth century, instructions to captains from the British Admiralty emphasized and repeated the message that no sailor was irreplaceable.^12^ The strategy of bringing along multiple ships and sailors had obviously high material and personal costs, a defining feature of premodern travel. While most ships did survive oceanic travel, many humans died along the way. Some 15 percent of early modern travelers from Europe to the East Indies never made it to their destination, most of them dying of disease on board or in shipwrecks.^13^ Arguably, it was only in the age of mass tourism that the idea of disposable ships and travelers went out of fashion. Once ships began to carry voyagers who paid for the experience, their risk of dying on the trip needed to be drastically reduced. In the twenty-first century, a time when the chance of airplane or railway catastrophes is approaching around one in ten million, the high value placed on passenger lives has turned vehicles into indispensable goods.^14^

If tourism made it impossible to rely on strategies of sending out multiple vessels to ensure the safe return of at least one, ships could still continue to rely on the alternative strategy of carrying extra timber, hemp, and other materials on board to fix broken masts and rudders, as well as torn rigging, together with the carpenters who could make the repairs.^15^ This strategy reduced the enormous costs of sending out multiple vessels at the same time, but it also necessitated the circulation of skilled workers and natural resources, which in itself was a large undertaking. The expenses of provisioning ships with repair materials were enormous, as the surviving account books of the Navy Board attest for England. Such strategies also survived into the early age of car travel. Before the establishment of a nationwide system of mechanics, early American drivers had to carry extra repair materials and tools so that they themselves could deal with breakdowns.^16^ The same strategy of redundancy is still operational in the airline industry where, for instance, most commercial aircraft are equipped and operate with four engines to ensure that the plane can fly even if three of its engines break down.^17^

If the two previous strategies of repair both relied on expertise and materials at the point of origin, a third strategy relied on resources and knowledge available at distant locations. In the age of sail, sailors frequently gathered timber to replace broken parts in the course of their journeys, chopping down trees wherever they went. Arguably, Columbus became assured of the possibility of establishing regular contact with the Americas when he discovered that the Caribbean contained enough timber to restock his ships for the return voyage. On November 24, 1492, a ship’s boy discovered pine groves “like thick and slender pokers, from which, he recognized, ships could be built and an infinite number of planks and masts for the largest ships in Spain.”^18^ Repair en route necessitated engagement with local populations, local vessels of transport, and local expertise of repair. The issue was not simply to access natural resources, but also to engage in conversation with people who could provide alternative vessels, perform complex repair work, and advise on the best way to go forward, sometimes by kidnapping indigenous pilots.^19^ These interactions literally transformed particular ships and even complete fleets. Such transformations are especially well-documented for the world of the early modern Indian Ocean.^20^ Several historians have pointed out, for instance, how, once European navies were established as a major player in the Indian Ocean, they immediately began to adapt to local conditions.^21^ In the case of the English Navy, the Bombay dockyards emerged as stations of ship repair in the early seventeenth century. There, expert Parsi shipwrights applied their knowledge toward repairing and rebuilding English ships, making them into hybrid vessels.^22^ Throughout the early modern period, English and other European naval officers debated vigorously whether one could trust foreign timber and foreign workers at such sites of ship repair. During the time of the Napoleonic wars, the English Navy seriously considered relocating its shipbuilding industry to Bombay and a heated debate ensued in Parliament. London shipwrights expressed strong concerns about competition from Bombay, claiming that Indian wood was low quality and Indian workers low skilled and untrustworthy.^23^ Local interactions about repair led to the development of travel infrastructure, but also to debates about the exact nature of skilled work and to racist diatribes about the trustworthiness of foreign people.^24^

Concerns about the reliability and control of workers are omnipresent in the history of transportation.^25^ Individual travelers and transportation companies still constantly oscillate between the strategy of relying on equipping vehicles and vessels with extra parts at the point of origin and that of relying on foreign expertise and materials. As Kevin Borg has documented, for instance, the history of American automobile repair is a history of tensions between the *bricolage* work of wayside mechanics, and car producers who have been making conscious efforts to standardize repair work and repairmen from the early 1930s onwards.^26^ The rise of computer diagnostics in the car industry is the story of the rise of centralized car repair, standardized labor techniques, and the alienation of the independent mechanic from his work.^27^ Controlling maintenance expertise and the location of repair became major issues for transportation companies on a global scale in the twentieth century. In Central and South America, the efforts of the United States to spearhead “Pan-American” projects of canal, railway, highway, and air travel foundered on project managers’ perceptions of intractably inhospitable local environments, labor, and social conditions.^28^ American journalists still rely on tropes of distrusting foreigners when they discover that aircraft maintenance is performed not in the United States, but in Central America or Asia.^29^

Yet a history of repair is not only a history of labor; it is also a history of environmental transformation. Strategies of carrying extra supplies placed the strain on the natural environment at the point of origin. As Karl Appuhn has documented, the shipbuilding industry of Renaissance Venice quickly resulted in the deforestation and environmental degradation of the neighboring Dalmatian coast, and the depletion of England’s forests was a major issue for the members of the Royal Society in Restoration England.^30^ The strategy of repairing vessels using natural resources available while traveling, in contrast, shifted the environmental consequences of transport away from Europe to distant locations. An engagement with the expertise of local populations also meant the destruction of non-European nature. As Richard Grove has shown, the Age of Explorations meant deforestation and concomitant soil erosion for the islands of the Caribbean, together with St Helena, Mauritius, and Cape Town.^31^ While historians and geographers often argue that repair and restoration can be panaceas against the large-scale production of waste that leads to climate change, it is important to realize that large-scale repair projects of transportation actually contributed to the transformation of the climate on a global scale.^32^

### Circulation, modernity, and producing knowledge of repair

The contributions to this special issue also reveal that a focus on repair and maintenance in transport networks provides a new angle from which to look at the global circulation of knowledge, arguably the most important topic in recent historiographies of science. In the past two decades, historians of science have explored in detail how natural knowledge is produced in circulation, arguing that “science is a form of communicative action” born in transit.^33^ Kapil Raj, Simon Schaffer, Lissa Roberts, and others have made the powerful claim that knowledge emerges when different populations establish contact zones where they can exchange information and commodities.^34^ In these accounts, the so-called Age of Discoveries, with its emphasis on long-distance maritime travel, is often taken to spur forced, unequal, but nonetheless productive exchanges between different populations. From Beijing to Calcutta and Potosí, a new set of connected non-European locations emerged as crucial sites for the construction of knowledge. Much of this literature has provided a reinvigorating reorientation for traditional narratives of the emergence of modern science. It has highlighted how natural knowledge emerged in hierarchical exchanges where credit, authorship, and even freedom were not granted to all participants. Yet even this literature has not fully engaged with the materialities of transport that facilitate these exchanges.^35^

This special issue claims that, if knowledge is produced in circulation, the material conditions of travel play a strong role in shaping it. Such an argument is inspired in part by the German tradition of media studies. As scholars such as Friedrich Kittler and Bernhard Siegert have argued, literature and culture are the aftereffects of developments and breakdowns in communication technologies.^36^ In recent years, Siegert and others have expanded this approach by exploring how transportation networks condition cultural and artistic production.^37^ Scholars in the maritime humanities such as Margaret Cohen and Steve Mentz have explored how oceanic travel often conditioned literature in the age of sail. As Mentz has argued, the shipwreck was the guiding literary metaphor of the early modern age, encompassing all the dangers and pitfalls of globalization while also exploiting the event’s potential for creating narrative tension.^38^ If Mentz is right, then early modern literature, from the *Lusiads* to Shakespeare’s *The Tempest* and *Robinson Crusoe*, is a side-effect of repair techniques gone wrong, just as, in Siegert’s account, modern literature is the side-effect of misfiring communications systems.

This special issue focuses, in contrast, on how natural knowledge is born when repair techniques do work. Transportation networks do not simply generate knowledge because they facilitate contact between different populations. Safe and secure transportation requires complex skills and knowledge of repair and maintenance, and much of the knowledge produced in long-distance networks focuses precisely on these practical concerns. Circulation produces the kinds of natural knowledge that facilitate further circulation, in other words. In recent years, scholars have already explored how much of the early modern mathematical, astronomical and cartographic sciences focused on solving the problems of navigation. Maps, astronomical instruments, and chronometers were all designed to ensure that ships reached their destination and found their way home.^39^ Yet a parallel history that focuses on the major turning points of repair techniques, and their relationship to scientific knowledge, has not been written. The contributions to this issue are marking the first steps toward writing such a story.

For the age of sail, for instance, a focus on repair can complement the current, extensive, and excellent literature on European efforts at optimizing naval architecture with a more global collection of studies on the natural history of timber and the parasites that attack it.^40^ As historians have begun to recognize, the shipworm *teredo navalis* was a major concern for early modern shipwrights, local experts, naval administrators, and natural philosophers because it bored into the hulls of ships and made them leak and sink.^41^ In the 1660s, Henry Oldenburg and Robert Boyle discussed the possibility that Indian pear-wood, a foreign tree, could be used to build ships instead of English timber because it was resistant to shipworm.^42^ The subsequent history of seventeenth- and eighteenth-century ship improvement presents a series of various failed or partially successful inventions to coat the hulls with protective paint or lead or copper sheathing, at enormous costs and expenses. As Frank James has documented, even nineteenth-century scientists were deeply involved in attempting to solve this problem. Humphry Davy turned his scientific prowess to practical purposes when he proposed adding zinc to the copper sheathing of ships, with catastrophic unexpected consequences that cost the British Navy more than a fortune before the project was abandoned.^43^ From the perspective of repair, moreover, the coming of the railway was not a radical departure from the age of sail. In India and across much of South and Southeast Asia, white ants and other parasites ravaged any type of transport infrastructure that relied on wood, be it the sleepers of train tracks or the planks of a ship.^44^ Technologies of transportation depended on each other to work, so that in China the first airplanes to make transnational journeys relied on gasoline that had been transported inland via the classic Silk Road transportation technology: camels. And a key phase in the early era of manned flight was the construction of seaplanes or “flying boats,” which did not need runways to land or take off, adapting technologies of oceangoing navigation to the air.

These previous examples from a long and unwritten history of repair, together with the stories told in this special issue, emphasize how transportation technologies rely on using local resources to solve breakdowns in local conditions. They hew closely to the vast historiography on how technologies are transformed when they are adapted to new locales. It is probably not an accident that Rudolf Mrazek’s *Engineers of a Happy Land*, one of the groundbreaking works that explored how technology becomes transformed when moved across the globe, focused primarily on transportation when examining how Western technologies were adopted in Indonesia by different actors for widely differing purposes.^45^ As things break down and are repaired, they slowly become different. A historiography of repair is also always a historiography of invention and innovation elsewhere.

Such an approach to transportation also problematizes historical claims about the key role of transportation techniques in the emergence of modernity. A variety of scholars such as Daniel Headrick, John Urry, John Law, and Bruno Latour have made powerful arguments about mobility and the establishment of long-distance networks as the key issue in the emergence of modernity.^46^ Much of this scholarship tends to consider transportation as a story of innovations enacted in Europe and North America, and to make the assumption that the development of modernity is a story of how European ships, trains, airplanes, and cars could conquer the globe without undergoing adaptation to local circumstances. Even actor network theory, which is highly attentive to the problems of translation and adaptation, appears to offer a similar narrative when it comes to explaining the emergence of modern Western power or the circulation of vessels and information. The sixteenth-century Portuguese navy was one of the major case studies for John Law’s theories of controlling networks, which he developed in tandem with Bruno Latour’s concept of immutable mobiles.^47^ Law argued that the success of Portuguese colonizers relied on their ability to keep their sailors and ships identical, faithful, and obedient across long distances, just as Latour’s inscriptions maintained their identity throughout their travels. Yet recent historians have presented the story of Portuguese colonization in drastically different colors.^48^ By the end of the sixteenth century, neither the ships nor the crew of the Portuguese were truly Portuguese. A focus on repair reveals that, in order to travel, survive, and succeed, things need to maintain their flexibility and mutability. To paraphrase Anna Tsing, whose work on globalization is key for the arguments of these essays, transportation is made possible by the productive work of friction.^49^ Every vehicle is a ship of Theseus. If modernity is about global networks of circulation (and if there is such a thing as modernity), it is a shape-shifting modernity that shows a different face at every site of repair. At each locale, a new kind of knowledge relies on the expertise of different craftsmen and the availability of different natural resources to keep global circulation in perpetual motion.

The challenges that transportation technologies pose to actor network theory, and to theories of modernity, resonate with the arguments of the emerging field of critical infrastructure studies. As Cymene Howe and others have pointed out, infrastructures are by nature paradoxical and liable to escape any strict designations. They are both material, bearing the traits of large technological systems, and philosophical, laden with semiotic and aesthetic meaning: in the words of Brian Larkin, they are “objects that create the grounds on which other objects operate.”^50^ They deteriorate despite the fact that their purpose is typically to reproduce structures across time and space; they are fragile and require retrofitting, even as they are designed to withstand the test of time; and they mitigate risk but also generate it, often because of their anthropocentric design.^51^ In the context of transportation technologies, one might think of infrastructure as including, for instance, not only a particular airplane, but also the airports, fields, runways, hangars, and factories in which airplanes are loaded and unloaded, repaired, and retrofitted, as well as the licensing regulations for pilots, air traffic control protocols, and regulatory laws applied by authorities.^52^ Infrastructures of transportation, and the provisions they made for maintenance and repair, articulated and embodied the local forms of knowledge that globalization yielded. They also often provided the means by which transportation technologies changed local environments, labor markets, and cultures.^53^

## Conclusion

The contributions to this issue provide the first steps toward a long-term history of repair over the past millennium. They epitomize the complex political, social, and technological expertise that repair en route requires, and how these encounters have shaped the development of natural knowledge and modern science along the way. As Pepijn Brandon and Marten Dondorp show, shipbuilding and ship repair were large-scale industries that needed complex natural resources and large groups of skilled workers.^54^ As a result, the establishment of complex repair facilities across the globe, and the transfer of shipbuilding technologies from one site to another, were highly difficult enterprises that tended to fail. Building and repairing ships in a cost-efficient manner, accessing the right resources at the right prices, and employing suitable skills of labor management required much-needed expertise in management.

Yet, despite the difficulties of transporting repair knowledge, ship repair and shipbuilding did happen across the globe. In her contribution, Bronwen Everill offers a case study of the establishment of a cluster of successful repair and building facilities in Freetown, Sierra Leone, in West Africa. She shows how Africans and Euro-Americans participated in the exchange of technologies related to shipbuilding and repair. Her story reveals how the presence of a local community of West African boat builders was essential for the success of establishing sites of repair for oceangoing ships built elsewhere; it also reveals the significance of canoe-building in coastal trade for Africans, Europeans, and Americans.^55^ Her article therefore serves as a reminder that, as Euro-American sailors traveled across the globe, they did not only rely on the ships they brought with them; they also adopted and adapted local methods of transportation.

If Sierra Leone shows how to make repair infrastructures work, Sara Caputo’s paper on voyages of discovery in the late eighteenth century British Empire reveals the limits of these infrastructures.^56^ As Caputo shows in great detail, port cities across the world did not only provide the opportunity to fix ships, but they could also be sites of danger where inexperienced pilots could wreck a vessel and seamen could be poached. Even then, repair was a necessity. Caputo argues that the discovery ships of the British Navy, arguably the most powerful naval power in the eighteenth century, needed constant care and attention to travel at excruciatingly slow speeds.

Similarly, in Mary Brazelton’s study of Republican China, the limitations of technical infrastructures were constant concerns for authors and administrators who sought to establish and promote aviation ventures.^57^ Airplanes could not fly across China without the construction of airfields and airports, weather observation and reporting systems, or radio communications networks. Brazelton argues that in the 1920s and 1930s, the education of skilled mechanics, engineers, and other staffers therefore became a priority for a number of transnational aviation ventures, because technical infrastructures required capable labor corps – thereby suggesting that histories of transportation maintenance and repair cannot be easily disentangled from histories of the labor associated with these activities.

Examining transport technologies across a variety of local environments also reveals the frequent coexistence and competition of different technologies of transportation over long periods of time. A global history of travel does not offer a linear history of unitary modernity. In his contribution, Stefan Tetzlaff shows how the coexistence and competition of motor cars and bullock carts in twentieth-century India shaped networks of transportation there.^58^ He suggests that those who advocated for *and* those who protested against the rise of motor transport in India collectively over-emphasized the transformative power of automobiles upon agrarian society. Tetzlaff presents a scenario in which the juxtaposition of old and new technologies, their designers, and their users reflected the competition and collaboration of various underlying ideas of modernity and technoscientific knowledge.

Taken together, the essays of this special issue reveal the immense amount of work that goes into transportation repair across the globe. Production and repair sites have long been unequally distributed across the globe, contributing to the production and reproduction of social and political inequalities. While the story of repair presents a history of science and technology that moves beyond the traditional confines of Europe and North America, it does not ignore the fact that practices of repair did not eliminate social and political hierarchies. Ships and places of repair were always sites of complex and mostly hierarchical labor organization, leading to the unequal distribution of profit and the unequal attribution of credit across the centuries. When repair was performed at a distance, it not only contributed to the production of hybrid types of knowledge, but it could also fuel the nationalist and racist tendencies of colonial powers. While repair and maintenance are often presented as ecological alternatives to an economy of waste, this essay has argued instead that fixing ships and other vehicles often had disastrous consequences for island and port environments in the early modern period.^59^ And by the dawn of the twenty-first century, global transportation and its sites of maintenance have become not only the foundational building blocks of a connected and networked society based on exchanges of knowledge, but also one of the main causes of human-made climate change. Repair provides a means to consider the checkered and connected histories of globalization across the past several centuries, and to reconsider how the material culture of transportation matters for the rewriting of that history.

## Footnotes

1. Robert Parthesius, Karen Millar, and Bill Jeffery, “Preliminary Report on the Excavation of the 17th-Century Anglo-Dutch East-Indiaman *Avondster* in Bay of Galle, Sri Lanka,” *International Journal of Nautical Archaeology* 34 (2005): 211–32.

2. Kevin Michaels, “MRO Industry Outlook,” April 28, 2016, <www.aeromontreal.ca/download/fca8adddfff353/01-MRO+Industry+Outlook_Kevin+Michaels.pdf> (October 10, 2019).

3. Cátia Antunes, “European Shipbuilding and Ship Repairs outside Europe: Problems, Questions and Some Hypotheses,” *International Journal of Maritime History* 31/3 (2019): 456–64; see also the other articles in the same issue.

4. David Edgerton, *The Shock of the Old: Technology and Global History since 1900* (London: Profile Books, 2006).

5. Andrew L. Russell and Lee Vinsel, *The Innovation Delusion: How Our Obsession with the New Has Disrupted the Work That Matters Most* (New York: Penguin Random House, 2020); Andrew L. Russell and Lee Vinsel, “After Innovation, Turn to Maintenance,” *Technology and Culture* 59 (2018): 1–25; Steven J. Jackson, “Rethinking Repair,” in Tarleton Gillespie, Pablo Boczkowski, and Kirsten Foot (eds.), *Media Technologies: Essays on Communication, Materiality and Society* (Cambridge, MA: MIT Press, 2014), pp.221–40; Jerome Denis, Alessandro Mongili, and David Pontille, “Maintenance and Repair in Science and Technology Studies,” *Tecnoscienza* 6 (2015): 5–15. See also the Maintainers’ website at <www.themaintainers.org> (October 10, 2019).

6. Stephen Graham and Nigel Thrift, “Out of Order: Understanding Repair and Maintenance,” *Theory, Culture & Society* 24 (2007): 1–25.

7. For instance, see the otherwise excellent Philip S. Bagwell, *The Transport Revolution from 1770* (London: Batsford, 1974).

8. John Urry, *Mobilities* (Cambridge: Polity Press, 2007); James der Derian (ed.), *The Virilio Reader* (Oxford: Blackwell, 1998).

9. Harry Collins, *Changing Order: Replication and Induction in Scientific Practice* (London: SAGE, 1985); Steven Shapin and Simon Schaffer, *Leviathan and the Air-Pump: Hobbes, Boyle and the Experimental Life* (Princeton: Princeton University Press, 1985). On replication in a maritime context, see Michael Wintroub, *The Voyage of Thought: Navigating Knowledge across the Sixteenth-Century World* (Cambridge: Cambridge University Press, 2017).

10. For such exceptions, see Jonathan Lamb, *Scurvy: The Disease of Discovery* (Princeton: Princeton University Press, 2016); Johannes Fabian, *Out of Our Minds: Reason and Madness in the Exploration of Central Africa* (Berkeley: University of California Press, 2000); Sue Ann Prince, *Stuffing Birds, Pressing Plants, Shaping Knowledge: Natural History in North America, 1730–1860* (Philadelphia: American Philosophical Society, 2003); Christopher M. Parsons and Kathleen S. Murphy, “Ecosystems under Sail: Specimen Transport in the 18th Century French and British Atlantics,” *Early American Studies* 10 (2012): 503–39. While these material conditions have already been examined in detail in the literature on the Middle Passage, the histories of science, technology, and medicine have not yet developed a sustained conversation with historians of slavery on this topic. See Stephanie Smallwood, *Saltwater Slavery: A Middle Passage from Africa to American Diaspora* (Cambridge, MA: Harvard University Press, 2007); Sowande M. Mustakeem, *Slavery at Sea: Terror, Sex, and Sickness in the Middle Passage* (Urbana: University of Illinois Press, 2016); and for the application of these lessons to the history of knowledge, Judith Carney, *Black Rice: The African Origins of Rice Cultivation in the Americas* (Cambridge, MA: Harvard University Press, 2002).

11. Francesca Lardicci (ed.), *A Synoptic Edition of the Log of Columbus’ First Voyage* (Turnhout: Brepols, 1999).

12. See, for instance, the instructions for the *Beagle*: Katherine Anderson (ed.), *The Narrative of the Beagle Voyage, 1831–1836* (London: Pickering & Chatto, 2012), Vol. I, p.13.

13. J. R. Bruijn, F. S. Gaastra, and I. Schöffer, *Dutch-Asiatic Shipping in the 17*th and 18th *Centuries* (The Hague: Martinus Nijhoff, 1987), Vol. I, p.163.

14. R. Amalberti, “The Paradoxes of Almost Totally Safe Transportation Systems,” *Safety Science* 37 (2001): 109–26.

15. Anatauarii Leal-Tamarii and Emmanuel Nantet, “Aboard a Rudderless Ship: Replacing Stern Rudders Mid-Voyage in the English and French Navies, 1750–1850,” *The Mariner’s Mirror* 107 (2021): 6–22.

16. Kevin Borg, *Auto Mechanics: Technology and Expertise in Twentieth-Century America* (Baltimore: Johns Hopkins University Press, 2007).

17. John Downer, “When Failure Is an Option: Redundancy, Reliability and Regulation in Complex Technical Systems,” *LSE Centre of Analysis of Risk and Regulation Discussion Paper No. 53*, 2009. http://eprints.lse.ac.uk/36537/1/Disspaper53.pdf

18. Lardicci, *A Synoptic Edition*, p.74 (note 11). For similar concerns along the Pacific, see Margaret Steven, *Trade, Tactics and Territory: Britain in the Pacific 1783–1823* (Carlton: Melbourne University Press, 1983) and Virginia Dellino-Musgrave, *Maritime Archaeology and Social Relations: British Action in the Southern Hemisphere* (New York: Springer, 2006).

19. Joyce Chaplin, *Round about the Earth: Circumnavigation from Magellan to Orbit* (New York: Simon and Schuster, 2012), pp.32–3.

20. For a comparative examination of shipbuilding technologies in Europe and Asia, and the limitations of these interactions between these traditions, see Richard W. Unger, “The Technology and Teaching of Shipbuilding, 1300–1800,” in Maarten Prak and Jan Luiten van Zanden (eds.), *Technology, Skills, and the Pre-Modern Economy in the East and the West: Essays Dedicated to the Memory of S. R. Epstein* (Leiden: Brill, 2013), pp.161–204.

21. Vitor Luis Gaspar Rodrigues, “The ‘Easternisation’ of the Portuguese Fleets in the Asian Seas during the 16th Century: Causes and Consequences,” in Lotika Varadarajan (ed.), *Gujarat and the Sea* (Vadodara: Darshak Itihas Nidhi, 2011), pp.221–50; K. M. Mathew, *History of Portuguese Navigation in India (1497–1600)* (Delhi: Mittal Publications, 1988).

22. R. A. Wadia, *The Bombay Dockyard and the Wadia Master Builders* (Bombay: Thakur Nivas, 1957).

23. House of Commons, *Minutes of the Evidence Taken before the Select Committee of the House of Commons on Petitions Relating to East-India-Built Shipping* (London: House of Commons, 1814).

24. Dániel Margócsy, “Technology Transfer, Ship Design, and Urban Policy in the Age of Nicolaes Witsen,” in Bert de Munck and Antonella Romano (eds.), *Knowledge and the Early Modern City: A History of Entanglements* (New York and London: Routledge, 2019), pp. 149–170.

25. Simon Schaffer, “‘The Charter’d Thames’: Naval Architecture and Experimental Spaces in Georgian Britain,” in Lissa Roberts, Simon Schafer, and Peter Dear (eds.), *The Mindful Hand: Inquiry and Invention from the Late Renaissance to Early Industrialisation* (Amsterdam: Koninklijke Nederlandse Akademie van Wetenschappen, 2007), pp.279–305; Robert C. Davis, *Shipbuilders of the Venetian Arsenal: Workers and Workplace in the Preindustrial City* (Baltimore: Johns Hopkins University Press, 2007).

26. Borg, *Auto Mechanics* (note 16).

27. As such, the story resembles that described more generally by Harry Braverman, *Labor and Monopoly Capital* (New York: Monthly Review Press, 1974).

28. Ricardo Donato Salvatore, “Imperial Mechanics: South America’s Hemispheric Integration in the Machine Age,” *American Quarterly* 58 (2006): 662–91.

29. James B. Steele, “The Disturbing Truth about How Airplanes are Maintained Today,” *Vanity Fair* (November 9, 2015), <www.vanityfair.com/news/2015/11/airplane-maintenance-disturbing-truth> (October 10, 2019).

30. Karl Appuhn, *A Forest on the Sea: Environmental Expertise in Renaissance Venice*. Baltimore: Johns Hopkins University Press, 2009.

31. Richard H. Grove, *Green Imperialism: Colonial Expansion, Tropical Edens and the Origins of Environmentalism, 1600–1860* (Cambridge: Cambridge University Press, 1995).

32. For such arguments, see, for instance, Jackson, “Rethinking Repair” (note 5), and Simon Werrett, *Thrifty Science: Making the Most of Materials in the History of Experiment* (Chicago: University of Chicago Press, 2019).

33. James A. Secord, “Knowledge in Transit,” *Isis* 95 (2004): 654–72, 663.

34. Kapil Raj, *Relocating Modern Science: Circulation and the Construction of Knowledge in South Asia and Europe, 1650–1900* (New York: Palgrave Macmillan, 2007); Simon Schaffer, Lissa Roberts, Kapil Raj, and James Delbourgo (eds.), *The Brokered World: Go-Betweens and Global Intelligence, 1770–1820* (Sagamore Beach, MA: Science History Publications, 2009).

35. But see Richard Sorrenson, “The Ship as Scientific Instrument in the Eighteenth Century,” *Osiris* 11 (1996): 221–36.

36. Friedrich Kittler, *Discourse Networks 1800/1900* (Stanford: Stanford University Press, 1990); Friedrich Kittler, *Gramophone, Film, Typewriter* (Stanford: Stanford University Press, 1999); Bernhard Siegert, *Relays: Literature as an Epoch of the Postal System* (Stanford: Stanford University Press, 1999).

37. Bernhard Siegert, “The Chorein of the Pirate: On the Origin of the Dutch Seascape,” *Grey Room* 57 (2007): 26–47; Franco Moretti, *Atlas of the European Novel, 1800–1900* (London: Verso, 1999); Wolfgang Schivelbusch, *The Railway Journey: The Industrialization of Time and Space in the Nineteenth Century* (Berkeley: University of California Press, 1977). For the sciences, see Peter Galison, *Einstein’s Clocks, Poincare’s Maps: Empires of Time* (New York: W. W. Norton, 2003).

38. Margaret Cohen, *The Novel and the Sea* (Princeton: Princeton University Press, 2012); Steve Mentz, *Shipwreck Modernity: Ecologies of Globalization, 1550–1719* (Minneapolis: University of Minnesota Press, 2015).

39. Karel Davids, *Zeewezen en wetenschap. De wetenschap en de ontwikkeling van de navigatietechniek in Nederland tussen 1585 en 1815* (Amsterdam: De Bataafsche Leeuw, 1986); Maria Portuondo, *Secret Science: Spanish Cosmography and the New World* (Chicago: University of Chicago Press, 2009); Simon Schaffer, “Newton on the Beach: The Information Order of the *Principia Mathematica*,” *History of Science* 47 (2009): 243–76; Rebekah Higgitt and Richard Dunn, *Finding Longitude: How Ships, Clocks and Stars Helped Solve the Longitude Problem* (Glasgow: Collins, 2014).

40. Larrie D. Ferreiro, *Ships and Science: The Birth of Naval Architecture in the Scientific Revolution, 1600–1800* (Cambridge, MA: MIT Press, 2007).

41. On shipworms, see Adam Sundberg, “An Uncommon Threat: Shipworms as a Novel Disaster,” *Dutch Crossing: Journal of Low Countries Studies* 40 (2016): 122–38; Lodewijk C. Palm, “Achtergronden van het paalwormenonderzoek, 1730–1870,” *Tijdschrift voor de Geschiedenis der Geneeskunde, Natuurwetenschappen, Wiskunde en Techniek* 13 (1990): 88–101.

42. Henry Oldenburg to Robert Boyle, March 13, 1666 (London: The Royal Society Archives, inv. EL/OB/49).

43. Frank A. J. L. James, “Davy in the Dockyard: Humphrey Davy, the Royal Society and the Electro-chemical Protection of the Copper Sheathing of His Majesty’s Ships in the mid 1820s,” *Physis* 29 (1992): 205–25.

44. Rohan Deb Roy, “White Ants, Empire and Entomo-Politics in South Asia,” *The Historical Journal* 63 (2020), 411–436. On labor and maintenance in this context, see Ian J. Kerr, “The Transfer of Railway Technologies and Afro-Asian Labor Processes within the British Empire,” *HoST – Journal of *History of Science* and Technology* 12 (2018): 31–74.

45. Rudolf Mrazek, *Engineers of a Happy Land: Technology and Nationalism in a Colony* (Princeton: Princeton University Press, 2002). On railways in India, see Ian Kerr, *Building the Railways on the Raj, 1850–1900* (Delhi: Oxford University Press, 1995).

46. Daniel Headrick, *The Tools of Empire: Technology and European Imperialism in the Nineteenth Century* (Oxford: Oxford University Press, 1981); Urry, *Mobilities* (note 8); John Law and John Hassard (eds.), *Actor Network Theory and After* (Oxford: Blackwell, 1999); Bruno Latour, *Reassembling the Social: An Introduction to Actor-Network-Theory* (Oxford: Oxford University Press, 2005). For a helpful critique of theories of modernity, see Gurminder K. Bhambra, *Connected Sociologies* (London: Bloomsbury, 2014).

47. John Law, “On the Methods of Long-Distance Control: Vessels, Navigation, and the Portuguese Route to India,” in John Law (ed.), *Power, Action and Belief: A New Sociology of Knowledge?* (New York: Routledge, 1986), pp.234–63; John Law, “Technology and Heterogeneous Engineering: The Case of Portuguese Expansion,” in Wiebe Bijker, Thomas Parke Hughes, and Trevor J. Pinch (eds.), *The Social Construction of Technological Systems* (Cambridge, MA: MIT Press, 1987), pp.111–34; Bruno Latour, “Visualisation and Cognition: Drawing Things Together,” in H. Kuklick (ed.), *Knowledge and Society: Studies in the Sociology of Culture Past and Present 6* (Greenwich: Jai Press, 1990), pp.1–40.

48. Sanjay Subrahmanyam, *The Portuguese Empire in Asia, 1500–1700: A Political and Economic History*, 2nd ed. (Malden, MA: John Wiley and Sons, 2012); Zoltán Biedermann, *(Dis)connected Empires: Imperial Portugal, Sri Lankan Diplomacy, and the Making of a Habsburg Conquest in Asia* (Oxford: Oxford University Press, 2018).

49. Anna Tsing, *Friction: An Ethnography of Global Connection* (Princeton: Princeton University Press, 2005).

50. Brian Larkin, “The Politics and Poetics of Infrastructure,” *Annual Review of Anthropology* 42 (2013): 327–43.

51. See “Critical Infrastructure Studies” at cistudies.org; Cymene Howe, Jessica Lockrem, Hannah Appel, et al., “Paradoxical Infrastructures: Ruins, Retrofit, and Risk,” *Science, Technology, & Human Values* 41 (2016): 547–65; Daniela Rosner and Morgan Ames, “Designing for Repair? Infrastructures and Materialities of Breakdown,” *CSCW 2014*: *Proceedings of the 17th ACM Conference on Computer Supported Cooperative Work & Social Computing* (2014): 319–331.

52. Langdon Winner, *The Whale and the Reactor: A Search for Limits in an Age of High Technology* (Chicago: University of Chicago Press, 1986); Penelope Harvey, Casper Bruun Jensen, and Atsuro Morita, “Introduction: Infrastructural Complications,” in Penelope Harvey, Casper Bruun Jensen, and Atsuro Morita (eds.), *Infrastructures and Social Complexity: A Companion* (Abingdon: Routledge, 2016), pp.1–22; Paul Edwards, “Infrastructure and Modernity: Force, Time, and Social Organization in the History of Sociotechnical Systems,” in Philip Brey, Arie Rip, and Andrew Feenberg (eds.), *Technology and Modernity: The Empirical Turn* (Cambridge, MA: MIT Press), pp.185–225.

53. For example, the Grand Canal in China (Jinghang da yunhe 京杭大运河), the oldest manmade canal system in the world, dates to the Sui dynasty (581–618 CE). Throughout the many centuries of its existence, it has grown into a network of canals yielding particular forms of ships, each with its own culture of repair. It has also produced economies of production sustained by the canal, inspired a wide variety of literary and cultural production, and drastically altered the environmental history of China by providing a stable route to feed the arid north. See Mark E. Lewis, *China’s Cosmopolitan Empire: The Tang Dynasty* (Cambridge, MA: Harvard University Press, 2012), pp.21–5.

54. Pepijn Brandon and Marten Dondorp, “Nodes of knowledge, managing transfer: Shipbuilding and repair during the transformation from sail to steam,” *History of Science* 61 (2023): 19–39.

55. Bronwen Everill, On a global history of maritime technologies, see also Ruth Barnes and David Parkin, *Ships and the Development of Maritime Technology on the Indian Ocean* (New York: Routledge, 2015); Lynn Harris, *Patroons and Periaguas: Enslaved Watermen and Watercraft of the Lowcountry* (Columbia, SC: University of South Carolina Press, 2014); Jason McGraw, *The Work of Recognition: Caribbean Colombia and the Postemancipation Struggle for Citizenship* (Chapel Hill, NC: UNC Press, 2014). Bronwen Everill, “‘For the Services of Shipwrights, Coopers, and Grummettas’: Freetown‘s Ship Repair Cluster in Nineteenth-Century Sierra Leone,” *History of Science* 61 (2023): 60–76.

56. Sara Caputo, “Exploration and Mortification: Fragile Infrastructures, Imperial Narratives, and the Self-Sufficiency of British Naval ‘Discovery’ Vessels, 1760–1815,” *History of Science* 61 (2023): 40–59.

57. Mary Augusta Brazelton, “Aviation infrastructures in the Republic of China, 1920-37,” *History of Science* 61 (2023): 102–120.

58. Stefan Tetzlaff, “Contested “automobility”: Peasants, townsfolks, and infrastructures of road transport in interwar central and western India (c. 1919–39),” *History of Science* 61 (2023): 77–101.

59. On the related issue of waste disposal in the Global South, regarding ships, see N. Gregson, M. Crang, F. Ahamed, N. Akhter, and R. Ferdous, “Following Things of Rubbish Value: End-of-Life Ships, ‘Chock-Chocky’ Furniture and the Bangladeshi Middle Class Consumer,” *Geoforum* 41 (2010): 846–54; for a more general overview, see Nate Millington and Mary Lawhon, “Geographies of Waste: Conceptual Vectors from the Global South,” *Progress in Human Geography* 43/6 (2019): 1044–63.

